# Supplementary material for: Real higher-order Weyl photonic crystal
Source: Nat Commun. 2023 Oct 20;14:6636. doi: 10.1038/s41467-023-42457-2 (PMC10587095; doi:10.1038/s41467-023-42457-2)
Supplement: Supplementary file 1 — Supplementary Information [file 41467_2023_42457_MOESM1_ESM.pdf]

Supplementary Information of
Real higher-order Weyl photonic crystal

Yuang Pan[1,2,3,4,#], Chaoxi Cui[5,6,#], Qiaolu Chen[1,2,3,4,#], Fujia Chen[1,2,3,4], Li Zhang[1,2,3,4], Yudong Ren[1,2,3,4], Ning Han[1,2,3,4], Wenhao Li[1,2,3,4], Xinrui Li[1,2,3,4], Zhi-Ming Yu[5,6,*], Hongsheng Chen[1,2,3,4,*], Yihao Yang[1,2,3,4,*]

[1]Interdisciplinary Center for Quantum Information, State Key Laboratory of Extreme Photonics and Instrumentation, ZJU-Hangzhou Global Scientific and Technological Innovation Center, Zhejiang University, Hangzhou 310027, China.

[2]International Joint Innovation Center, The Electromagnetics Academy at Zhejiang University, Zhejiang University, Haining 314400, China

[3]Key Lab. of Advanced Micro/Nano Electronic Devices & Smart Systems of Zhejiang, Jinhua Institute of Zhejiang University, Zhejiang University, Jinhua 321099, China

[4]Shaoxing Institute of Zhejiang University, Zhejiang University, Shaoxing 312000, China

[5]Centre for Quantum Physics, Key Laboratory of Advanced Optoelectronic Quantum Architecture and Measurement (MOE), School of Physics, Beijing Institute of Technology, Beijing 100081, China.

[6]Beijing Key Laboratory of Nanophotonics and Ultrafine Optoelectronic Systems, School of Physics, Beijing Institute of Technology, Beijing 100081, China.

*Correspondence to: (Y. Y.) yangyihao@zju.edu.cn; (Z. Y.) zhiming_yu@bit.edu.cn; (H. C.) hansomchen@zju.edu.cn

[#] These authors contributed equally: Yuang Pan, Chaoxi Cui, Qiaolu Chen.

Table of contents

Note 1. Tight-binding model 1

Note 2. Nontrivial Zak phase 4

Note 3. Simulated results of the evolution from spin-1 Weyl point to real higher-order Weyl points. 4

Note 4. Multiple perspectives of the 3D unit cell 6

Note 5. Experimental setup 7

Note 6. Topological surface states on the (010) surface 7

Note 7. First-principle calculations of the topological charges with Wilson loop method 8

Note 8. First-principle calculations of the generalized real Chern numbers using Wilson loop method 9

Note 9. Zak phase calculated by Wilson loop method 10

Note 10. Microwave properties of AlSi10Mg 10

Note 11. Convergence of simulation results 11

Supplementary References 13

Note 1. Tight-binding model

As investigated in Ref.[1], a symmetry-enforced spin-1 Weyl point (charge-2 triple point) located at the Γ point, in conjunction with a charge-2 Dirac point at R point, can be realized in a photonic crystal with space group (SG) No.198. The generators of the SG No. 198 encompass C_{2x}, $C_{3,111}$ and T operations. Intriguingly, we find that the $C_{3,111}$ symmetry plays a pivotal role in the formation of the spin-1 Weyl point[2], however, it is not necessary for the presence of the charge-2 Dirac point. Hence, upon breaking the $C_{3,111}$ symmetry of the photonic crystal, the charge-2 Dirac point remains intact, while the spin-1 Weyl point will be split into two real higher-order Weyl points.

The essential physics discussed here can be captured by a four-band tight-binding (TB) model, for which the structure is shown in Fig. S1. Each unit cell contains four atoms located at (0.45,0.2,0.2), (0.95,0.3,0.8), (0.05,0.7,0.3) and (0.55,0.8,0.7) positions, and each atom has a *s*-like orbit. The symmetry-allowed TB can be written as

$$H = t\begin{pmatrix} 0 & H_{12} & H_{13} & H_{14} \\ H_{12}^{\dagger} & 0 & H_{23} & H_{24} \\ H_{13}^{\dagger} & H_{23}^{\dagger} & 0 & H_{34} \\ H_{14}^{\dagger} & H_{24}^{\dagger} & H_{34}^{\dagger} & 0 \end{pmatrix}, \tag{1}$$

where t is the nearest hopping amplitude and

$$H_{ij} = e^{ir_{lmn}^{+\eta s}} + e^{ir_{lmn}^{-\eta s}} \ . \tag{2}$$

Here, $r_{lmn}^{\varepsilon\eta s} = (+\varepsilon\frac{k_l}{2} + \eta\frac{k_m}{10} + s\frac{2k_n}{5})$, with $\{l,m,n\} = \{x,y,z\}$, $\{\varepsilon,\eta,s\} = \pm$, $H_{12} = e^{ir_{xyz}^{++-}} + e^{ir_{xyz}^{-+-}}$,

$$H_{13} = e^{ir_{yzx}^{++-}} + e^{ir_{yzx}^{-+-}}, H_{14} = e^{ir_{zxy}^{++-}} + e^{ir_{zxy}^{-+-}}, H_{23} = e^{ir_{zxy}^{+++}} + e^{ir_{zxy}^{-++}}, H_{24} = e^{ir_{yzx}^{+--}} + e^{ir_{yzx}^{---}}, H_{34} = e^{ir_{xyz}^{+++}} + e^{ir_{xyz}^{-++}} \ .$$

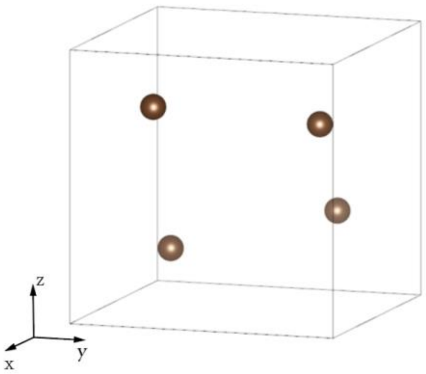

Fig.S1 The spatial structure of TB model. The cubic represents the unit cell. Each unit cell contains four atoms, which are represented by four spheres.

The band structure of this TB model is shown in Fig. S2a, along with the band representations at high-symmetry points. One can find that the four-band model exhibits a spin-1 Weyl point at the Γ point and a charge-2 Dirac point at the R point, just like the case in Ref.[1]. By introducing a

perturbation that breaks $C_{3,111}$ symmetry, the system's space group transitions from No. 198 to No. 19. The perturbation Hamiltonian may be written as

$$\Delta H = \delta_1 \begin{pmatrix} 0 & 0 & 0 & H_{14} \\ 0 & 0 & H_{23} & 0 \\ 0 & H_{23}^{\dagger} & 0 & 0 \\ H_{14}^{\dagger} & 0 & 0 & 0 \end{pmatrix} + \delta_2 \begin{pmatrix} 0 & 0 & H_{13} & 0 \\ 0 & 0 & 0 & H_{24} \\ H_{13}^{\dagger} & 0 & 0 & H_{34} \\ 0 & H_{24}^{\dagger} & 0 & 0 \end{pmatrix}. \tag{3}$$

The perturbed band structure is shown in Fig. S2b, where the spin-1 Weyl point at the Γ point indeed transforms into two C-1 Weyl points at z-axis. Simultaneously, the $k_z = 0$ plane become an insulator phase. We then show under which conditions the $k_z = 0$ plane is a real Chern insulator.

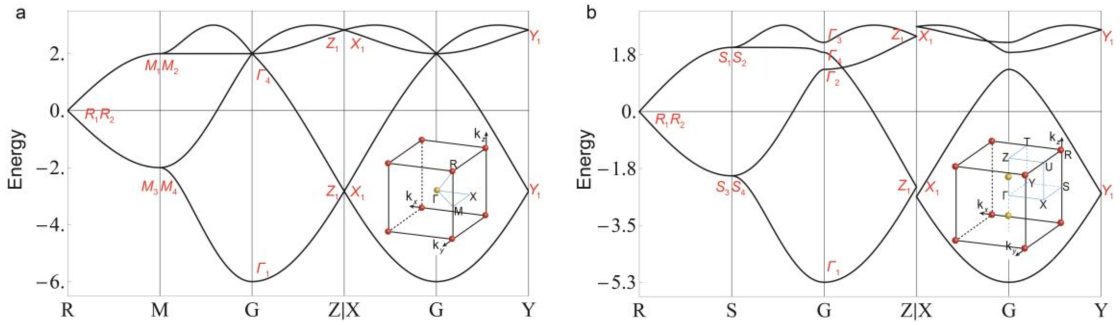

Fig. S2 a, Band structure of TB model (1) in space group No.198. **b,** Perturbed band structure. The inserts show the corresponding 3D Brillouin zone.

The C_{2z}-eigenvalue of each band representation at the four high-symmetry points: Γ, X, Y and M ($k_z = 0$ plane) of SG No. 198 and No. 19 are listed in Table S1. The C_{2z} eigenvalues are calculated from the tight-binding (TB) models. The C_{2z} eigenvalues of a state is calculated as follow. At a C_{2z} invariant point K, C_{2z} eigenvalues of nth band is defined as

$$\zeta_n = \langle \psi_{nK} | \hat{C}_{2z} | \psi_{nK} \rangle, \tag{4}$$

where $\hat{C}_{2z}$ is the C_{2z} operator and $|\psi_{nK}\rangle$ is the wave function of nth band at K. For the TB model, the Hamiltonian and wave functions are written in basis $\{|\varphi_{\alpha K}\rangle\}$,

$$|\varphi_{\alpha K}\rangle = \frac{1}{N}\sum_{i=1}^{N} e^{i\,\boldsymbol{K}\cdot(\boldsymbol{R}_i + \boldsymbol{r}_\alpha)} |\phi_{\alpha R_i}\rangle, \tag{5}$$

where $|\phi_{\alpha R_i}\rangle$ is the αth orbit in R_i cell. In this basis, wave function can be expanded as

$$|\psi_{nK}\rangle = \sum_{\alpha=1}^{N_{orbit}} c_{n\alpha} |\varphi_{\alpha K}\rangle, \tag{6}$$

$c_{n\alpha}$ is the αth element of nth eigenvector of Hamiltonian $H(k)$. By combining Eq. (1) and (3), one can get the expression of C_{2z} eigenvalue of nth band as

$$\zeta_n = \sum_{\alpha=1}^{N_{orbit}} \sum_{\beta=1}^{N_{orbit}} c_{n\beta}^{*} C_{2z\beta\alpha} e^{2i\boldsymbol{K}\boldsymbol{r}_\alpha} c_{n\alpha}, \tag{7}$$

where $C_{2z\beta\alpha}$ is the C_{2z} matrix under the basis of $\{|\phi_\alpha\rangle\}$. This can be rewritten into a compact matrix form

$$\zeta_n = c_n^{\dagger} C_{\beta\alpha} D_K c_n, \tag{8}$$

where $D_K = Diag\left(e^{2i\boldsymbol{K}\boldsymbol{r}_1}, e^{2i\boldsymbol{K}\boldsymbol{r}_2}, ...\right)$.

Noticed that there exists only one band representation for X (Y) point: X_1 (Y_1), which is doubly degenerate and possesses the C_{2z}-eigenvalue of (1,-1). Besides, there are two band representations for M (S) point, with C_{2z}-eigenvalues of (1,1) and (-1,-1). Hence, when the spin-1 Weyl point in SG No. 198 splits into two Weyl points and the $k_z = 0$ plane is gapped (see Fig. S2b), the lowest two bands of the TB model in Fig. S2b at the Γ point must be Γ_1 and Γ_2, ensuring that the $k_z = 0$ plane possesses a vanishing Chern number[3, 4]. This is because the Chern number (C) of $k_z = 0$ plane can be inferred by the C_{2z}-eigenvalue at Γ, X, Y and M points[3]

$$(-1)^C = \prod_{i \in occ} \zeta_i(\Gamma)\zeta_i(X)\zeta_i(Y)\zeta_i(M), \tag{9}$$

where ζ_i is C_{2z}-eigenvalue at Γ, X, Y and M points on the i-th band. Meanwhile, we find that for the TB model depicted in Fig. S2b the lowest two bands at the S point are $S_3 \oplus S_4$ with the C_{2z}-eigenvalue of (-1,-1). Then, according to the definition of real Chern number, one has $\nu_R = 1$ for the $k_z = 0$ plane. It should be noticed that for the TB model in Fig. S2b, the lowest two bands at S point also can be $S_1 \oplus S_2$ under suitable model parameter, and in such case the real Chern number of the $k_z = 0$ plane is zero.

Table. S1 C_{2z}-eigenvalue of the band representations at Γ, X, Y and M points. The Γ_4 of SG No. 198 is transformed into three different band representations: Γ_2, Γ_3 and Γ_4 of SG No. 19 upon symmetry breaking. The M point in SG No. 198 is labeled as S point in SG No. 19.

SG 198	Γ_1	Γ_4			X_1	Y_1	$M_1 \oplus M_2$	$M_3 \oplus M_4$
C_{2z}-eigenvalue	1	(-1,-1,1)			(1,-1)	(1,-1)	(1,1)	(-1,-1)
SG 19	Γ_1	Γ_2	Γ_3	Γ_4	X_1	Y_1	$S_1 \oplus S_2$	$S_3 \oplus S_4$
C_{2z}-eigenvalue	1	1	-1	-1	(1,-1)	(1,-1)	(1,1)	(-1,-1)

Since the $k_z = 0$ plane is a real Chern insulator, it will have corner states, which also can be understood from topological quantum chemistry (TQC)[5-8]. The $k_z = 0$ plane is a 2D system belonging to layer group No. 21. Analysis with TQC shows that, the 2D system is equivalent to that of two orbitals (Wannier centers) at the *2a* Wyckoff position: (0,0,z) and (1/2,1/2,-z). Notice that the *2a* site is not occupied by any atom of the TB model, as shown in Fig. S3. Generally, for a nanotube cutting through a Wannier center, it will have hinge states. However, it should be noticed that for certain

boundary conditions, the hinge states may coexist with the boundary states in the same energy range, which is not feasible for experimental observation. Specifically, for the model studied here, we find that a nanotube with a triangular cross section (see Fig. S3) is most suitable, which is not only convenient for experiment realization but also exhibits a clean hinge state.

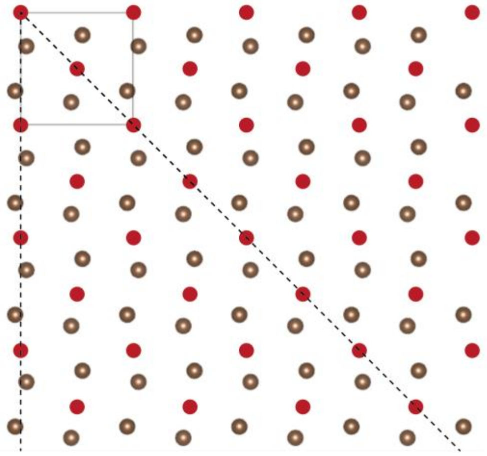

Fig. S3 Top view of the crystal structure of the perturbed TB model with the unit cell being denoted by the gray solid lines. The brown balls denote the atoms of the TB model, and the red balls are the Wannier centers obtained from the $k_z = 0$ plane. The dashed lines show the boundary of a nanotube.

Note 2. Nontrivial Zak phase

Due to the C_{2z} symmetry, the Zak phase along a line transverse the BZ

$$Z_{x(y)}(k_{y(x)}, k_z) = \frac{1}{2\pi}\int_0^{2\pi} \Omega_{n,xy}\, dk_{x(y)} \tag{10}$$

is quantized, for which the value can also be inferred from the C_{2z}-eigenvalue at two C_{2z}-invariant points[9, 10], as the C_{2z} symmetry is identical to a mirror symmetry for a one-dimensional system. Hence, the Zak phase Z_x and Z_y of all the $|k_z| < k_{wp}$ plane of SG No. 19 can be obtained as

$$(-1)^{Z_{x(y)}} = \prod\nolimits_{i\in occ} \zeta_i(\Gamma)\zeta_i(X(Y)) \tag{11}$$

According to C_{2z}-eigenvalue, one has $Z_x = Z_y = 1$ for all the $|k_z| < k_{wp}$ planes, which indicates the surface floating states between the two real higher-order Weyl points.

Note 3. Simulated results of the evolution from spin-1 Weyl point to real higher-order Weyl points.

First, we construct a photonic crystal featuring three C_2 screw symmetry along the x, y, z axis, and a C_3 rotational symmetry along the <111> axis. The 3D unit cell is shown in Fig. S4a. The simulated band structure is shown in Fig. S4b. As observed, the spin-1 Weyl point exists at Γ,

enforced by the screw symmetry and the $C_{3,111}$ symmetry. Subsequently, we break its $C_{3,111}$ symmetry by compressing the 3D unit cell along the y direction, the resulting crystal structure is illustrated in Fig. S4c, and the photonic band structure is displayed in Fig. S4d. Evidently, the spin-1 Weyl point splits into two real higher-order Weyl points. However, the crystal structure shown in Fig. S4c is excessively intricate for fabrication purposes. Therefore, we optimize the structure while maintaining its symmetry unaltered.

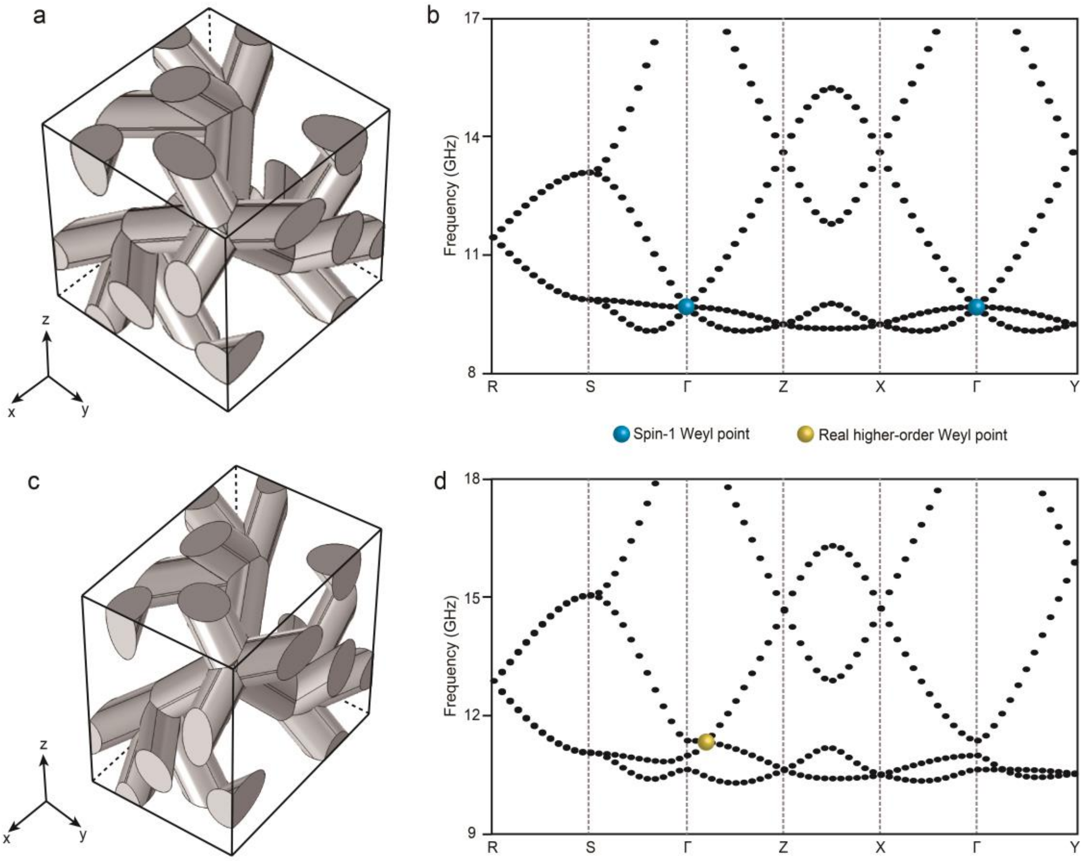

Fig. S4 a, Unit cell of 3D photonic crystal hosting a spin-1 Weyl point. **b,** Band structure of the photonic crystal shown in Fig. S4a. **c,** Unit cell of a 3D photonic crystal hosting two real higher-order Weyl points. **d,** Band structure of the photonic crystal shown in Fig. S4c.

Note 4. Multiple perspectives of the 3D unit cell

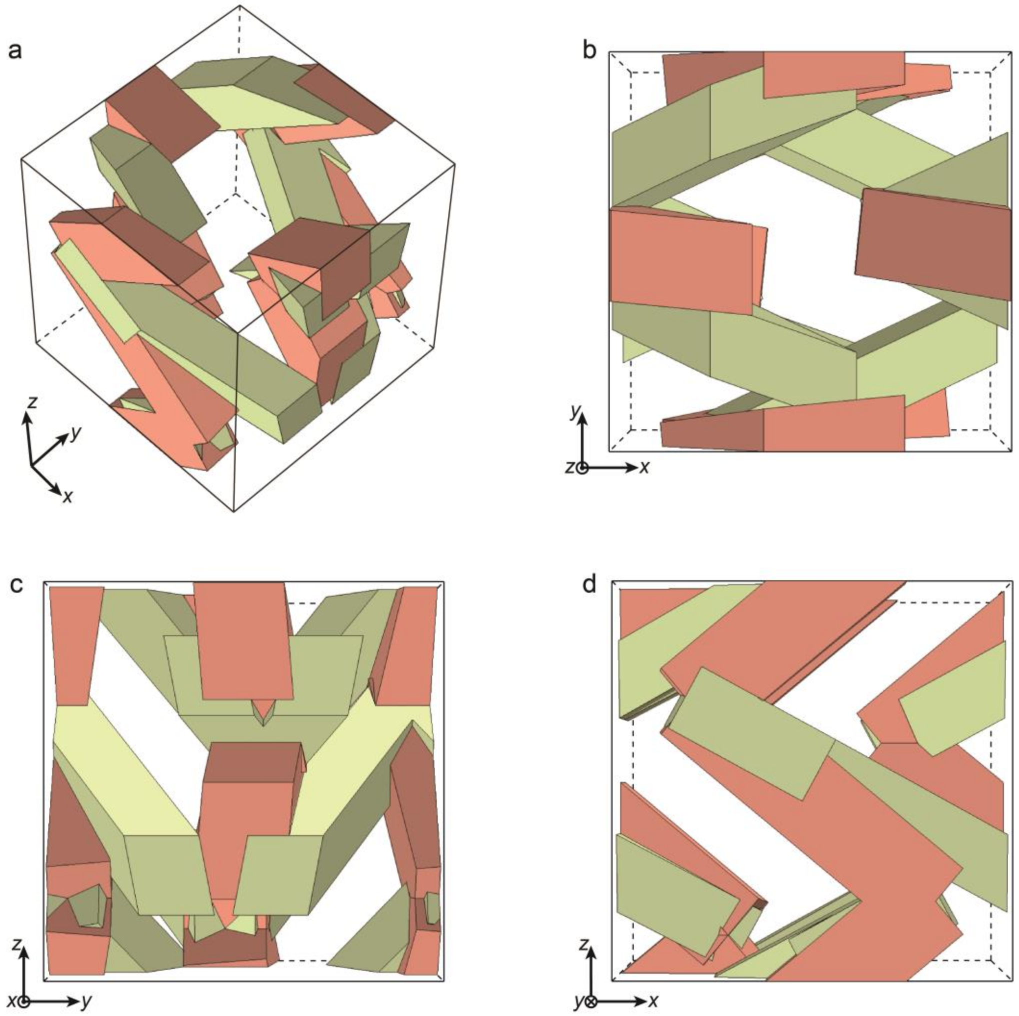

Fig. S5 a, Perspective view of the 3D unit cell. **b,** Top view of the 3D unit cell. **c, d,** Side view of the 3D unit cell.

Note 5. Experimental setup

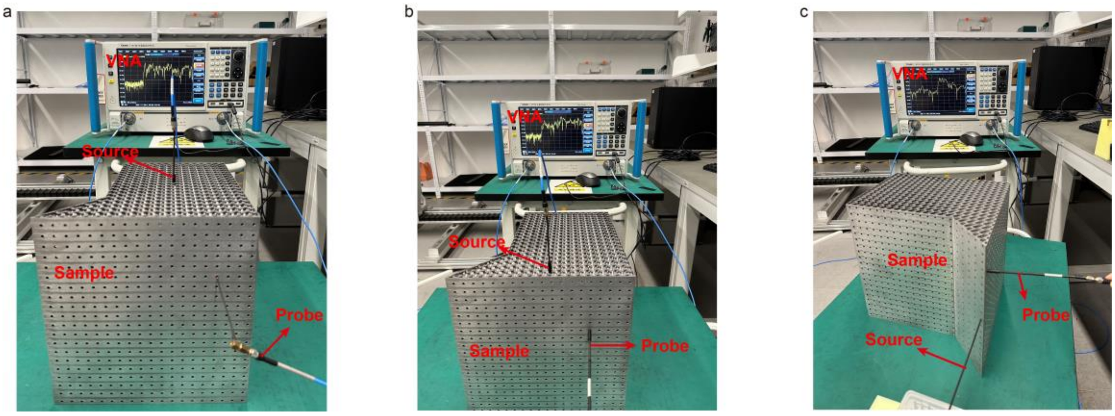

Fig. S6 Experimental setups. **a,** Experimental setups for measuring the bulk states, the source and probe are placed inside the sample. **b,** Experimental setups for measuring the surface states, the source and probe are placed in the surface of the sample. **c,** Experimental setups for measuring the hinge states, the source and probe are placed in the hinge of the sample

As displayed in Fig. S6, the experimental setup consists of a vector network analyzer (VNA), two cables, a source, a probe, and a fabricated sample. Fig. S6 a,b,c shows the experimental setup for measuring the bulk states, surface states, and hinge states respectively.

Note 6. Topological surface states on the (010) surface

The experimental configuration is depicted in Fig. S7a. The source is placed in the center of the (010) surface, and the probe is inserted into the sample to measure the field distributions on the surface. After applying a 2D transform to the measured field distribution, we obtain the surface dispersion, as illustrated in Fig. S7b. The measured surface dispersion along the high-symmetry line $\bar{\Gamma}-\bar{Z}-\bar{M}-\bar{X}-\bar{\Gamma}$ is displayed in Fig. S7b, and the Zak phase protected topological floating surface states is observed along $\bar{\Gamma}-\bar{Z}$ and $\bar{X}-\bar{\Gamma}$. The grey (green) dots represent the bulk (surface) states respectively. The color map measures the energy density. Fig. S7c-f show the Fermi arc surface states on the (010) surface of the photonic crystal.

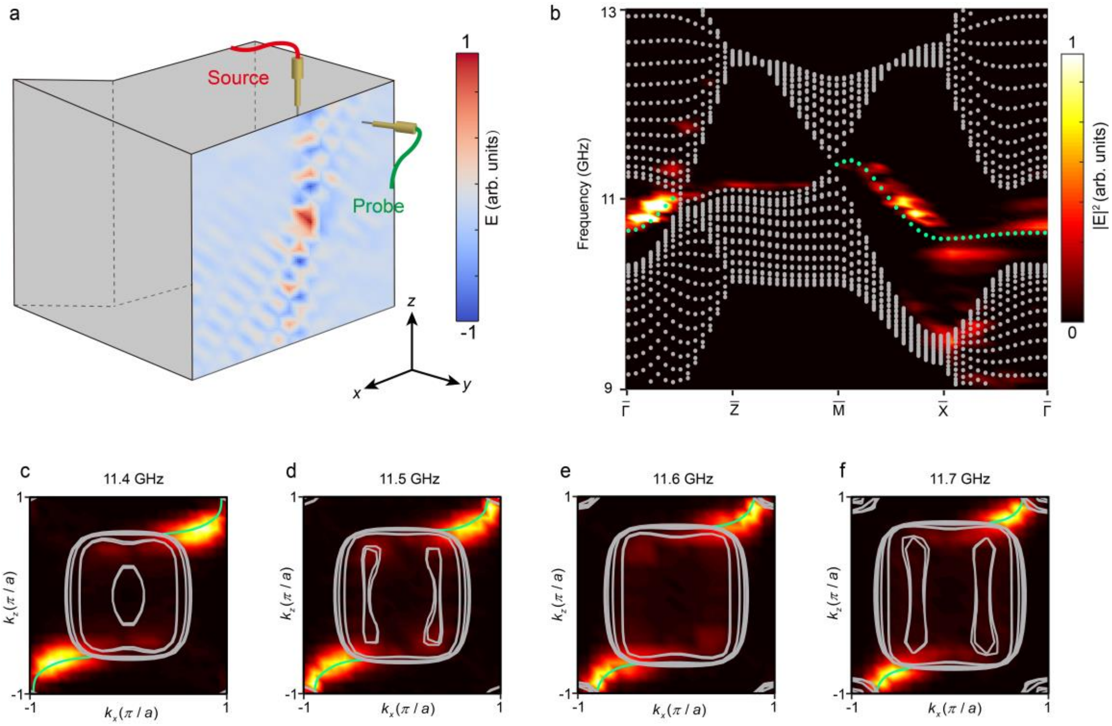

Fig. S7 a, Experimental set-up. **b,** Measured surface dispersion along the high-symmetry line $\bar{\Gamma}-\bar{Z}-\bar{M}-\bar{X}-\bar{\Gamma}$. The grey (green) dots represent the simulated bulk (surface) states respectively. The color map measures the energy intensity. **c-f,** Measured surface iso-frequency contours from 11.4 GHz to 11.7 GHz. The grey (green) curves represent the bulk (surface) dispersions.

Note 7. First-principle calculations of the topological charges with Wilson loop method.

In order to calculate the topological charges for the real Weyl point and the charge-2 Dirac point, we first track the evolution of Wannier centers on a sphere covering four kinds of band degenerate points. The sphere is discretized into a sequence of horizontal loops, from the north pole to the south pole of the sphere (i.e., the polar angle θ in spherical coordinates varies from 0 to π). Then the Berry phase along these horizontal loops can be numerically calculated by employing the Wilson loop method, in which the wavefunctions are extracted from the COMSOL Multiphysics calculations. The Wannier centers (ϕ) are simply the trace of the Berry phase.

Fig. S8 shows the Wannier centers for the lower band of the real higher-order Weyl point and the charge-2 Dirac point. For the real higher-order Weyl point and charge-2 Dirac point, the Wannier centers shift by 2π and -4π respectively, revealing the topological charge is 1 and -2, respectively.

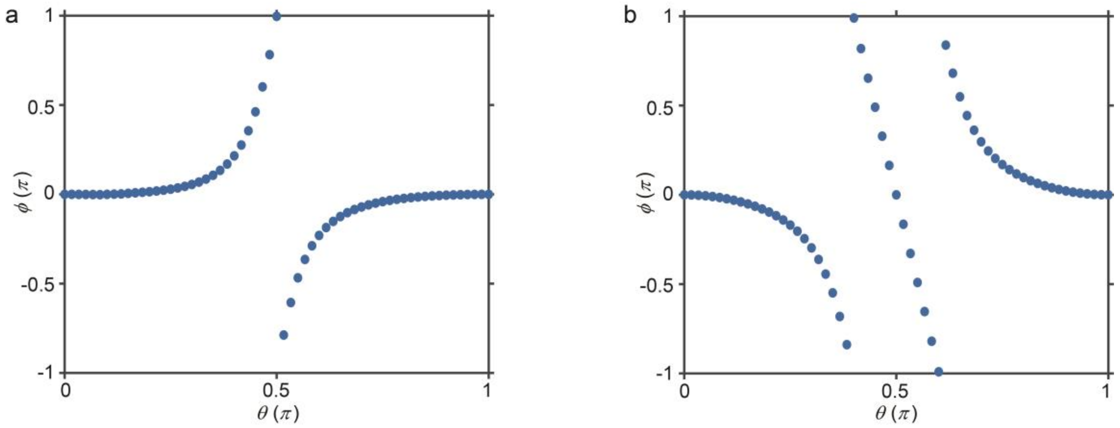

Fig. S8 Evolution of the Wannier centers on the spheres enclosing real Weyl point and charge-2 Dirac point, using Wilson loop method. a, Charge-1 real higher-order Weyl point. **b,** Charge-2 Dirac point.

Note 8. First-principle calculations of the generalized real Chern numbers using Wilson loop method

The generalized real Chern number ν_R is evaluated by using the Wilson loop method. For the k_z = 0 plane, we calculate the Berry phase along the <110> direction of the BZ, using the wavefunctions extracted numerically from the COMSOL Multiphysics calculations. The Wilson loop spectrum is shown in Fig. S9a, we observe that a single crossing exists at $\phi = \pi$, indicating the non-trivial generalized real Chern number $\nu_R = 1$. On the other hand, for the k_z = 0.75 π / a , Fig. S9b shows that the Chern number C = 1.

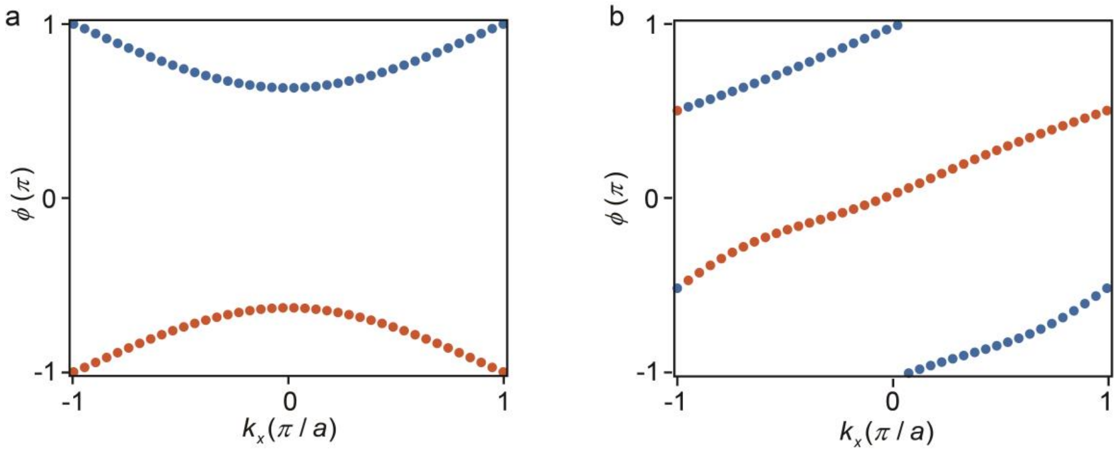

Fig. S9 a, Wilson loop spectrum calculated along <110> direction on the k_z = 0 plane. There exists one crossing point at $\phi = \pi$, indicating real Chern number $\nu_R = 1$. **b,** Wilson loop spectrum calculated along <110> direction on the k_z = 0.75 π / a plane, indicating Chern number C = 1.

Note 9. Zak phase calculated by Wilson loop method

The Zak phase is evaluated by using the Wilson loop method. For the $k_z = 0$ plane, we calculate the Berry phase along the <010> and <100> direction of the BZ, using the wavefunctions extracted numerically from the COMSOL Multiphysics calculations. The Wilson loop spectrum is displayed in Fig. S10, which indicates the Zak phase $Z_x = Z_y = 1$.

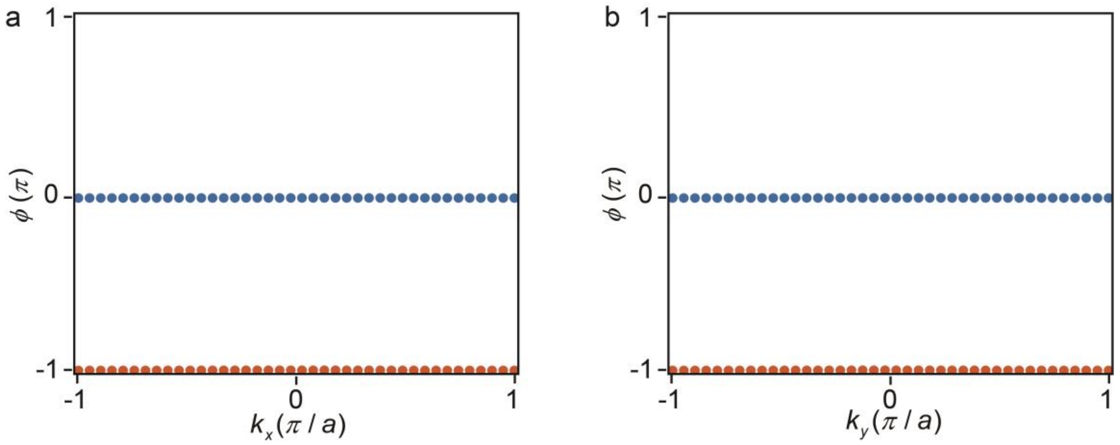

Fig. S10 a, Wilson loop spectrum calculated along the <010> direction on the $k_z = 0$ plane. **b,** Wilson loop spectrum calculated along the <100> direction on the $k_z = 0$ plane.

Note 10. Microwave properties of AlSi10Mg

At microwave frequencies, AlSi10Mg shows a very low metallic loss that can be ignored, just like other conventional metals, e.g., Au, Ag, and Cu. To confirm this, we designed a structure supporting surface waves. We fabricated two samples made of AlSi10Mg (see Fig. S11a) and copper (see Fig. S11b, copper is usually regarded approximately as a perfect electric conductor (PEC) at low frequencies), respectively. Using microwave measurement, we obtained the field distributions and the corresponding band structures of the surface waves (see Fig. S11c-e). We also compare our experimental results with simulated ones; in simulations, we set the metals as PEC. One can see that the measured results of the two metals are almost identical, and agree well with the simulated counterparts. All these results demonstrate that at microwave frequencies, AlSi10Mg has a negligible metallic loss, similar to the copper, and can be regarded approximately as PEC.

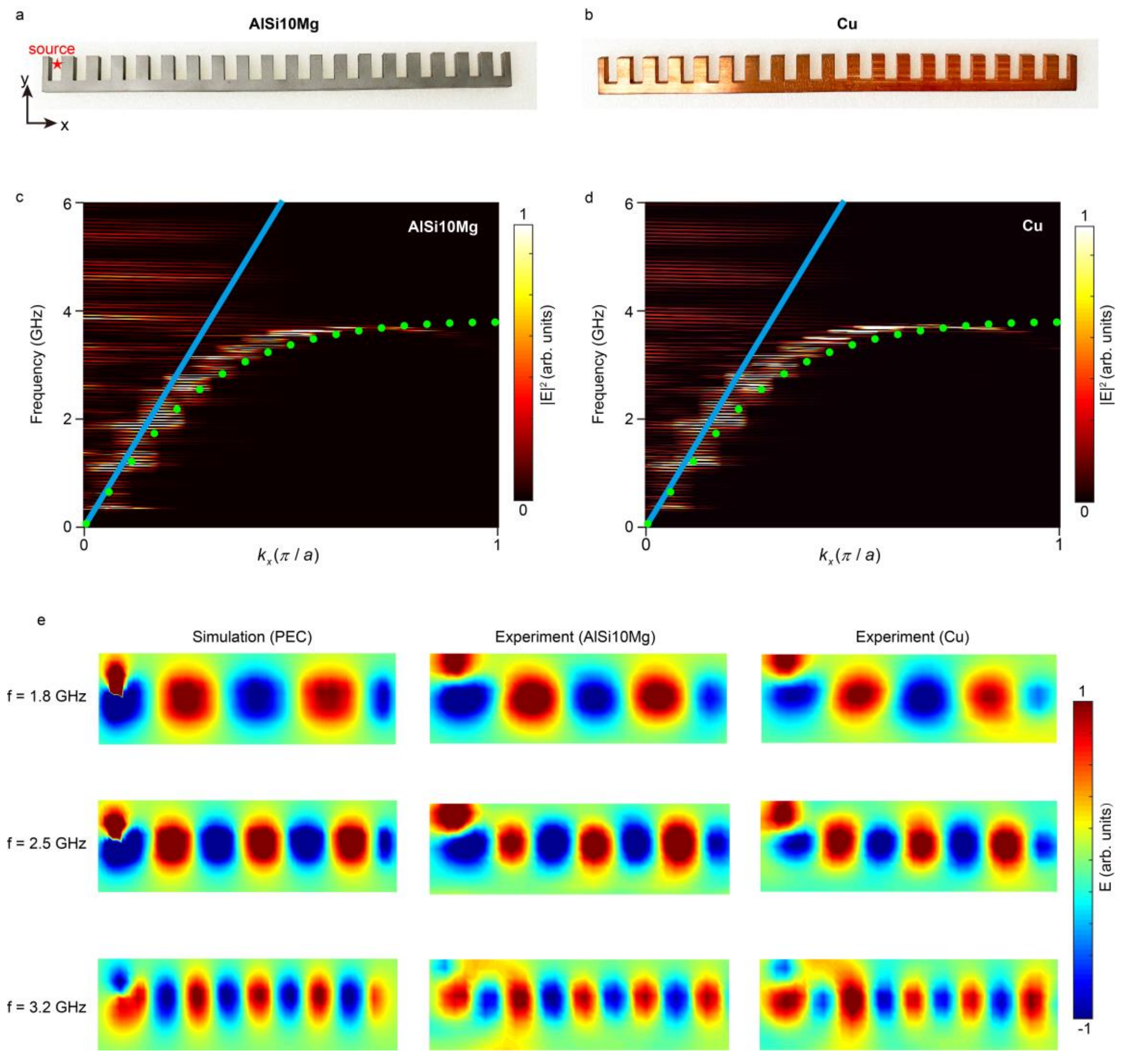

Fig. S11 a,b, Fabricated samples made of AlSi10Mg and Cu, respectively. The red star represents the source location. **c,d,** Measured dispersions of AlSi10Mg sample and Cu sample, respectively. The green dots display the simulated dispersion of a PEC structure, and the blue line shows the light line. **e,** Simulated and measured field distributions at different frequencies. The left, middle, and right columns represent the results of the structures made of PEC, AlSi10Mg, and Cu, respectively

Note 11. Convergence of simulation results

For the surface dispersion, we have changed the supercell size from 4 to 15 unit cells, and the simulated results are displayed in Fig. S12a-d. One can see that the surface dispersion is convergent when the size reaches 8 unit cells.

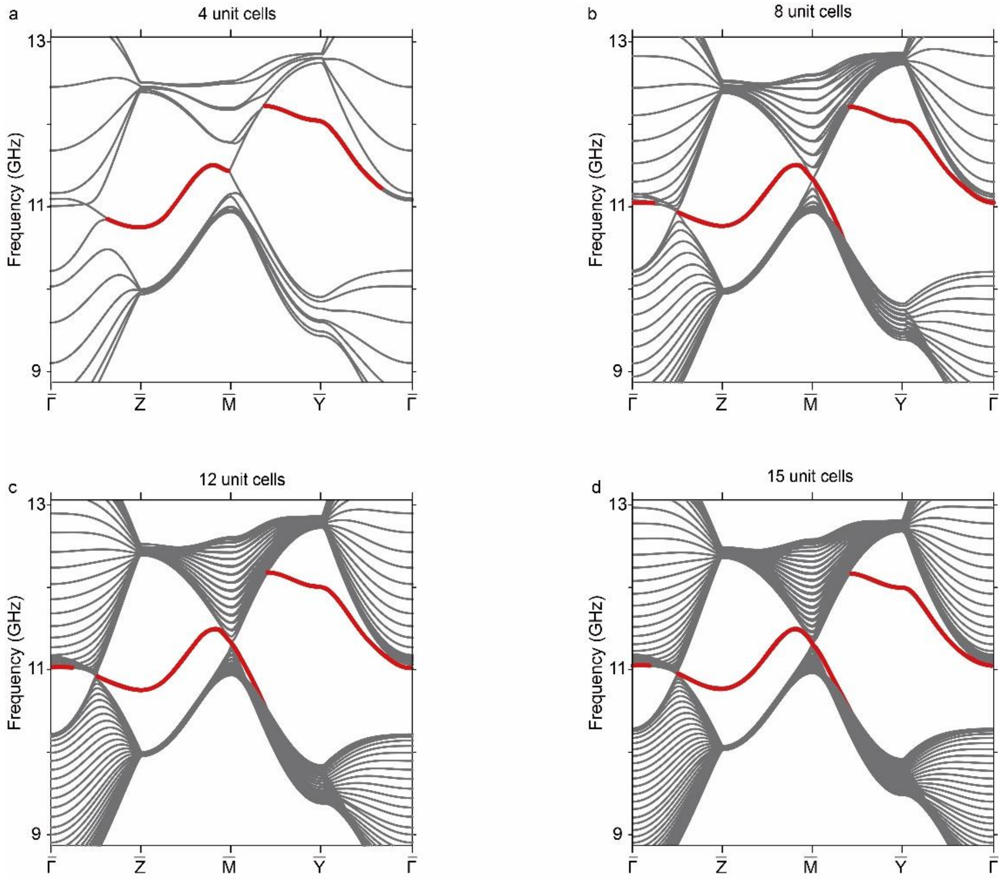

Fig. S12 The simulated surface dispersion when the supercell size changes from 4 to 15 unit cells. **a,** The simulated surface dispersion when the supercell consists of 4 unit cells. **b,** The simulated surface dispersion when the supercell consists of 8 unit cells. **c,** The simulated surface dispersion when the supercell consists of 12 unit cells. **d,** The simulated surface dispersion when the supercell consists of 15 unit cells. The red (grey) curves represent the surface (bulk) dispersions.

For the hinge dispersion, we change the supercell size from 3×3 to 11×11 unit cells, and the simulated results are displayed in Fig. S13a-d. One can see that the hinge dispersion is convergent when the size reaches 8×8 unit cells.

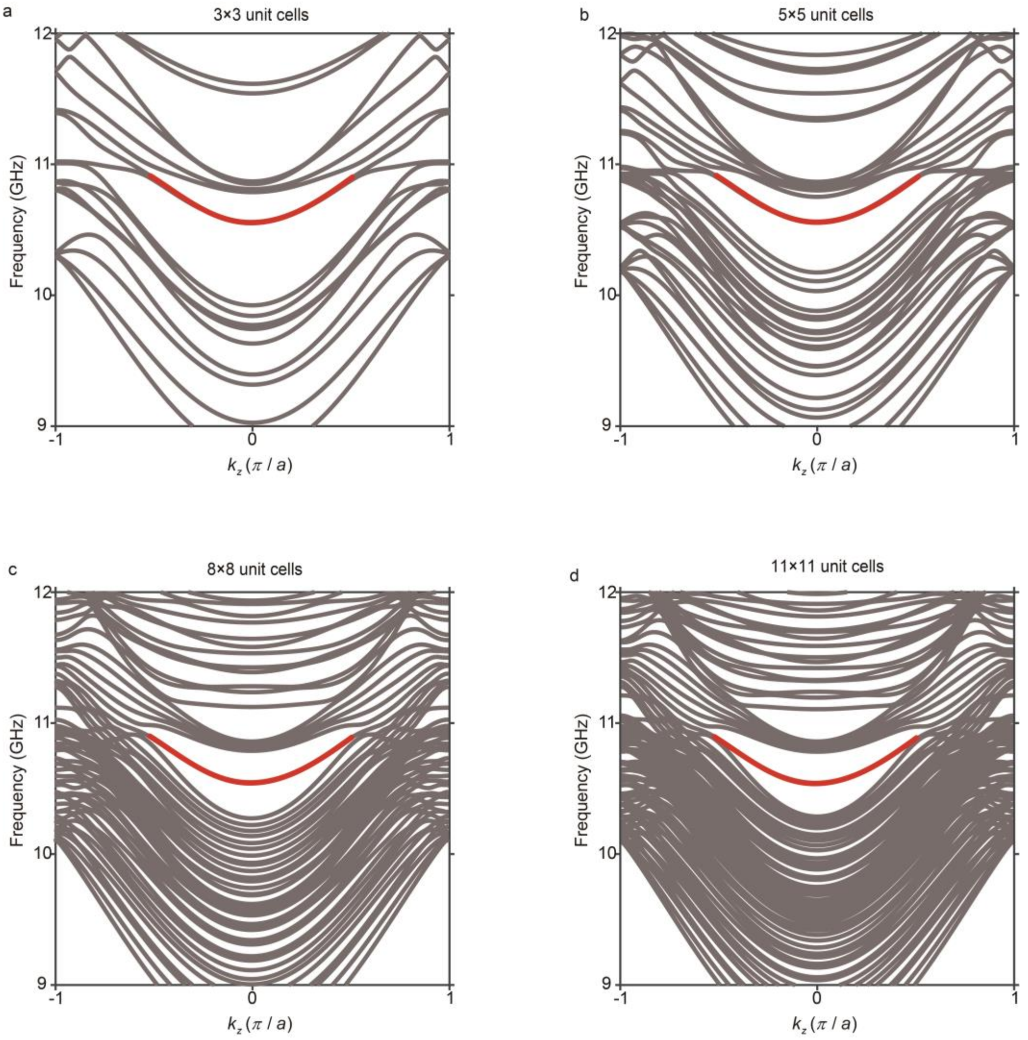

Fig. S13 The simulated hinge dispersion when the supercell size changes from 3×3 to 11×11 unit cells. **a,** The simulated hinge dispersion when the supercell consists of 3×3 unit cells. **b,** The simulated surface dispersion when the supercell consists of 5×5 unit cells. **c,** The simulated surface dispersion when the supercell consists of 8×8 unit cells. **d,** The simulated surface dispersion when the supercell consists of 11×11 unit cells. The red (grey) curves represent the hinge (bulk) dispersions.

Supplementary References

1. Yang, Y. et al. Topological triply degenerate point with double Fermi arcs. *Nat. Phys.* **15**, 645-649 (2019).
2. Feng, X., Wu, W., Huang, Y., Yu, Z.-M. & Yang, S.A. Triply degenerate point in three-dimensional spinless systems. *Phys. Rev. B* **104**, 115116 (2021).
3. Hughes, T.L., Prodan, E. & Bernevig, B.A. Inversion-symmetric topological insulators. *Phys. Rev. B* **83**, 245132 (2011).

4. Fang, C., Gilbert, M.J. & Bernevig, B.A. Bulk topological invariants in noninteracting point group symmetric insulators. *Phys. Rev. B* **86**, 115112 (2012).
5. Bradlyn, B. et al. Topological quantum chemistry. *Nature* **547**, 298-305 (2017).
6. Kruthoff, J., de Boer, J., van Wezel, J., Kane, C.L. & Slager, R.-J. Topological Classification of Crystalline Insulators through Band Structure Combinatorics. *Phys. Rev. X* **7**, 041069 (2017).
7. Po, H.C., Vishwanath, A. & Watanabe, H. Symmetry-based indicators of band topology in the 230 space groups. *Nat. Commun.* **8**, 50 (2017).
8. Song, Z.-D., Elcoro, L. & Bernevig, B.A. Twisted bulk-boundary correspondence of fragile topology. *Science* **367**, 794-797 (2020).
9. Fu, L. & Kane, C.L. Topological insulators with inversion symmetry. *Phys. Rev. B* **76**, 045302 (2007).
10. Chan, Y.H., Chiu, C.-K., Chou, M.Y. & Schnyder, A.P. Ca_3P_2 and other topological semimetals with line nodes and drumhead surface states. *Phys. Rev. B* **93**, 205132 (2016).
